# Design and Operation of Effective Landfills with Minimal Effects on the Environment and Human Health

**DOI:** 10.1155/2021/6921607

**Published:** 2021-09-06

**Authors:** Gulnihal Ozbay, Morgan Jones, Mohana Gadde, Shehu Isah, Tahera Attarwala

**Affiliations:** Department of Agriculture and Natural Resources, Delaware State University, Dover, DE 19901, USA

## Abstract

Totaling at 7.4 billion people, the world's population is rapidly growing, bringing along with it an increase in waste generation. The impact of this exponential increase in waste generation has resulted in the increased formation and utilization of landfills. In the present day, landfills are utilized to dispose of chemical, hazardous, municipal, and electronic wastes. However, despite their convenience, most landfills are improperly managed and face constant changes from the surrounding environment that interfere with their internal landfill processes. The objectives of this mixed review are to highlight the negative impacts landfills have on the environment and public health as well as outline the need for proper management practices to mitigate these effects. Inadequate management of landfills leads to issues concerning leachate collection and landfill gas (LFG) generation, which give rise to groundwater contamination and air pollution. This paper recognizes the disadvantages of utilizing landfills as the main disposal method by focusing on these two primary effects that improper management of landfills has on the environment and human health. Many experts have also reported that communities within close proximity to improperly managed landfills have an increased risk of health issues. Apart from implementing proper landfill management practices, it is important to develop solutions to reduce waste generation altogether. This review discusses some of the innovative methods implemented by other countries to reduce landfill waste and the production of greenhouse gases as well as possible steps individuals can take to minimize their ecological footprints.

## 1. Introduction

Waste management is a global issue, especially with the increased accumulation of solid waste every year [1]. In the 1990s, developed countries produced up to 800 kg of waste per capita, while developing countries produced a maximum of 200 kg per capita [2]. In 2010, the global solid waste production was estimated to be two billion tons [3]. Developed countries (the US, Canada, Japan, Australia, New Zealand, and Western Europe) accounted for half of this estimate. However, this estimate will reportedly change with urban population growth in developing countries. Further estimations show that by 2035, Asian and African countries will produce twice as much solid waste, while developed countries will produce less waste [3]. Nonetheless, current and future waste productions require effective landfill use, which varies globally. A landfill is defined as a well-engineered depression in the ground used for the disposal of solid waste. Landfills contain a variety of wastes, mainly consisting of municipal solid waste (MSW) or everyday single-use items such as packaging, grass clippings, furniture, clothing, bottles, food scraps, newspapers, appliances, paint, and batteries [4].

Municipal solid waste management differs throughout the world. For instance, zero percent of MSW in Sweden ends up in landfills, while in Bulgaria, almost 100 percent of their MSW ends up in landfills. In 2014, Europe overall sent 41% of collected MSW to landfills [1]. Unlike Europe, many Asian countries have other means of waste disposal such as open dumping and burning; therefore, any waste placed in landfills is done through unsanitary and unregulated methods. In the United States, over 50 percent of waste was landfilled in 2015 [1]. Although there are multiple means of waste disposal around the world, the use of landfills is one of the most common methods. This review will focus on landfill management in the US, with the majority of the data representing US waste generation.

According to the United States Environmental Protection Agency [4], every year the United States generates at least 254 million tons of waste. This translates to about two kg of waste generated per person, each day. Treatment of municipal solid waste in the United States (US) includes landfills (52.1%), recycling (25%), incineration or combusting with energy recovery (12.7%), and composting (10.1%) [4].

Relative proportions of these treatment methods from 1960 to 2017 are shown in Figure 6. According to the data, landfills are a more convenient waste disposal method, representing over 52% of waste disposed of in the US as of 2017. Our dependency on landfills has only continued to increase over the years due to an increase in population size. In 2000, the United States' population was 281.4 million, 308.7 million in 2010, and 323.1 million in 2016 [5]. With this population increase, the demand for manufactured products and materials grows, increasing waste generation as shown in Figure 7.

Consequently, different types of landfills exist in the US, each requiring different handling techniques and regulations for specific waste types. There are three main landfill types: industrial landfills, municipal solid waste landfills, and hazardous waste landfills. Industrial landfills process nonhazardous waste produced by industrial activities; municipal solid waste landfills (MSWLFs) mainly collect household and other general solid waste; and hazardous waste landfills process hazardous or toxic waste and are the most regulated and structured landfills. Each of these landfills have developed systems to manage the waste, which comprises liners, leachate collection, gas collection, drainage systems, runoff control, etc. There are also additional categories of landfills described in Section 2. Most wastes generated from our hospitals, schools, homes, and businesses are put into municipal solid waste landfills. Municipal solid waste landfills can also receive nonhazardous sludge, industrial solid waste, and construction debris, which can contribute to groundwater pollution, air pollution, and habitat destruction [4]. Many of these waste materials are nonbiodegradable and can sit in landfills for years without decomposing [4]. Landfills need to be continuously managed over long periods. Landfills are engineered so that they are located, designed, operated, and monitored to ensure compliance with federal regulations [4]. According to the Environmental Defense Fund [6], there are currently over 3,000 landfills in the United States.

Although landfills have satisfied the need for immediate waste disposal, this method is not ideal for long-term waste management and has multiple negative effects on the environment and public health. There are solid waste landfills that are well managed and designed as part of the integrated waste management system and protect the environment from contaminants that may be present in the solid waste stream; however, that is not always the case. Most landfills still contribute to many environmental issues including groundwater contamination from leachate generation and emission of greenhouse gases from landfill gas (LFG) generation [7]. Landfills can also render surrounding soil and land unusable. With the population increase and the inevitable growth in waste production, efforts toward proper waste management and waste reduction in the United States must be made to prevent further environmental damage [8].

The objectives of this mixed review are to (i) highlight the negative impacts landfills have on the environment and public health, (ii) outline the need for proper management practices to mitigate these effects, and (iii) evaluate possible solutions to manage waste generation, through the use of past case studies and research conducted on this topic and data provided by the Environmental Protection Agency. Bias is handled in this review by including multiple credible sources that support the claim that landfills have negative impacts on the environment and public health and by including both statistical data and photo evidence that further demonstrate this issue. This review will bridge the research gap from previous studies by comparing the practice and effectiveness of multiple landfill types as well as evaluating innovative methods to reduce landfill waste.

## 2. Design and Regulations of Landfills

In the US, all municipal solid waste landfills, industrial landfills, and hazardous landfills are expected to meet minimum national criteria under the “Resource Conservation and Recovery Act (RCRA)” to ensure the protection of human health and the environment [9]. An industrial waste landfill for disposal of nonhazardous industrial waste or commercial solid waste is regulated by RCRA subtitle D wastes. Specific regulations for handling various types of hazardous wastes are contained under subtitle C of RCRA in title 40 of the code of federal regulations (CFR): part 264 for permitted facilities and part 265 for interim status facilities [9]. These standards, in general, apply to owners and operators of landfill facilities across the United States of America.

There are multiple different landfill designs, each with their own separate processes and characteristics. Each landfill design has varying degrees of sustainability. These landfills include open dump landfills, controlled landfills, engineered landfills, and sustainable landfills.

### 2.1. Open Dump Landfills

**Figure 1:**
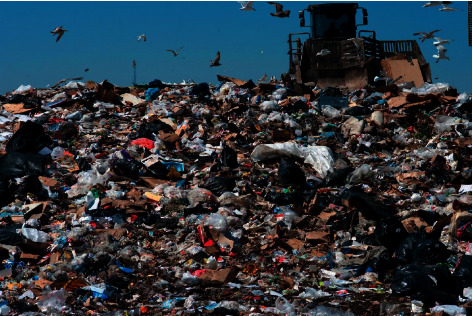
An open dump landfill (reprinted from HelpSave Nature, https://helpsavenature.com/).

Open dumping is a common practice in many developing countries around the world and is defined as a method of disposal of solid wastes indiscriminately without planning or control mechanisms. About 70% of countries around the world use “open dumping” as a method of disposal of municipal solid waste. Since these open dumpsites are not regulated, they are susceptible to open burning, scavengers, disease vectors, and elements [10]. The characteristics of these open dumpsites include lack of planning and control of dumpsites, inadequate or lack of regulation of types of wastes entering the site, waterlogging and leaching resulting in water pollution, open defecation by the public, lack of confinement of waste body, and uncontrolled burning of waste materials leading to air pollution. Open dump landfills are prohibited in the US [11]. A typical open dumpsite is shown in Figure 1.

These open dumpsites have no proper engineering design and therefore have no groundwater protection or drainage controls. Environmental risks posed by these open dump landfills need to be investigated to determine remedial actions on whether to close or upgrade the open dump to a controlled landfill. Environmental impact assessments (EIAs) should include flaws in site location (floodplains or groundwater), depth of existing open dumpsite and degree of compaction, variability of wastes within the site, and potential for mining decomposed organic materials [12].

### 2.2. Controlled Landfills

Controlled landfills are one level above open dump landfills, as controlled landfills are subject to basic control mechanisms such as the presence of an authority figure on site, control of vehicular movement and access to landfill, and basic waste handling techniques to ensure control and consolidation of the total body of wastes. At these sites, there is an installation of preliminary drainage control measures and a lack of uncontrolled burning of waste, and scavenging and foraging animals are minimized.

Although controlled landfills are more regulated than open dump landfills, they are still not viable since they do not conform with the fundamental principles of waste compaction and covering. Typical operational procedures include limiting the working face area, installation of litter barrier, and provision of daily cover. Waste volume is subject to control, as well as drainage systems and water quality.

### 2.3. Engineered Landfills

**Figure 2:**
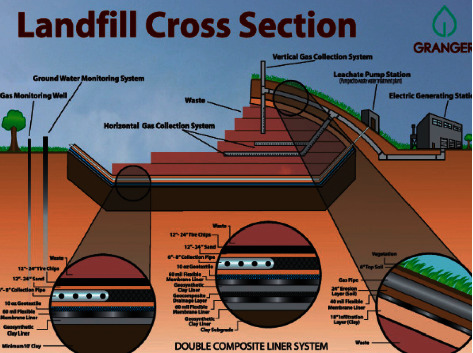
Mid-Michigan Engineered landfill design, https://www.grangernet.com./

Engineered landfills are disposal sites that are constructed through planning and adoption of engineering techniques that ensure control of waste and avoidance of surface water through the installation of well-designed and well-constructed surface drainage. Other characteristics include excavation and spreading of soil materials to cover the body of wastes, compacting of wastes into smaller layers, removal of leachate from wastes into lagoons or similar structures, venting of landfill gas out of wastes, and most importantly planned isolation of landfills from surrounding geology. These modern landfills are based on the concept of isolating landfills from the environment for proper stabilization of wastes and rendering them innocuous through biological, chemical, and physical treatments. An engineered landfill is represented by the Mid-Michigan landfill design in Figure 2.

Engineered landfills are often referred to as sanitary landfills due to the high standard of waste disposal. Sanitary landfills require a protected bottom where trash is buried in layers and compressed as a compact solid to ensure the safety of accumulated waste and ease of decomposition. The design, construction, and development of these landfills require sufficient planning from inception to its after-use stage. Location siting, construction, and operational requirements are much more stringent than other types of landfills. Thus, sanitary or engineered landfills are considered to have the least impact on public health and the environment [13].

### 2.4. Sustainable Landfills

The major driver of engineered or sanitary landfills has been the prevention of waste saturation to minimize the likelihood of leachate leaking into the surrounding ground. This approach has led to a very slow rate of waste degradation, with a projected stabilization period in the order of hundred years. However, degradation can be accelerated in principle by the controlled circulation of fluids through the waste and thus operating such engineered landfill as a bioreactor. This approach is more sustainable with regard to airspace, processes, control, and product utilization with minimal negative impacts on the environment and human health.

**Figure 3:**
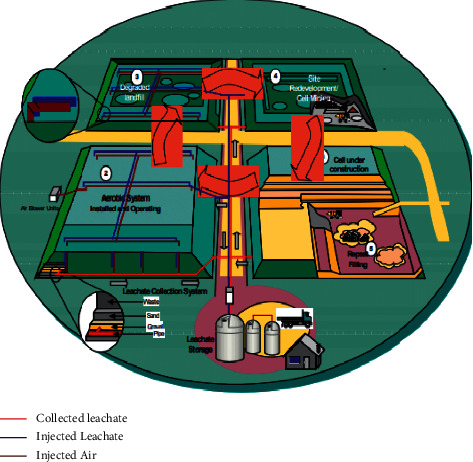
Aerobic biocell designed by the Environmental Control System, Inc., in South Carolina (2001).

Sustainable landfills often have two different approaches with regard to parameters that control chemical and biological processes such as water content, temperature, microflora, and compaction rates. These led to anaerobic bioreactors and aerobic biocells [14, 15]. Anaerobic bioreactors are similar in design to an engineered landfill with the following basic difference in their operational practice: a built-in leachate collection and recirculation system to enhance waste stabilization, geomembrane liners, a gas collection system, and final cover. Using this system, the methane gas that is predominantly produced can be collected, purified, and sold. Aerobic biocell systems utilize air circulation to maximize the rate of decomposition of waste. This latter system generates carbon dioxide as a preferred gas. A sustainable landfill utilizing an aerobic biocell design built by the Environmental Control System, Inc. (2001), in South Carolina is shown in Figure 3.

Stabilized waste in this system has limited methane gas and odor production, generates less harmful leachate capable of impacting groundwater, and ensures that the landfill recovers valuable airspace paving the way for a recycle (reusable) and sustainable landfill system.

## 3. Environmental and Human Health Risks from Landfills

A decision as to whether a landfill should be closed, rehabilitated, or remediated involves technical investigations and environmental impact assessments (EIAs). This requires a wide range of consultations with interested parties, especially the adjacent communities. A typical risk assessment process includes a set of logical, systemic, and well-defined activities that provide sound and unambiguous identification, measurement, quantification, and evaluation of the risks associated with landfills. Potential adverse effects to public health and the environment require evaluations of waterborne and airborne pollutants, assessment of the number of people affected by these pollutants, characteristics of wastes associated with the landfills, size of the landfill defined by the total amount of solid waste disposed of, and potential health conditions and psychological effects to public health.

Some simple quantification tools for risk assessments include hazard potential rating developed by Saxena and Bhardwaj [16] for an upgrade of existing municipal solid waste. Attributes are often grouped and weighed and therefore assigned weightage of attribute (W_i_) such that the total weight was 1000. Each attribute is also measured in terms of sensitivity index (Si) on a scale of 0 to 1 to facilitate computation of cumulative scores called risk index (RI). Computed risk indexes are used to classify landfills for their environmental and public health impact. The RI is calculated according to

(1) $$\begin{array}{c} \text{RI}=\sum W_{i}S_{i}, \end{array}$$

where W_i_ is the weightage of the i-th variable ranging from 0 to 1000; S_i_ is the sensitivity index of the i-th variable ranging from 0 to 1; and RI is the environmental risk index.

Landfills with high RI scores indicate a greater risk to human health and require immediate remedial measures. Landfills with low RI scores indicate low sensitivity and significant environmental impacts.  Table 1 summarizes some of the tools that can be used for risk index assessments.

### 3.1. Environmental Impacts of Landfills

#### 3.1.1. Leachate Infiltration into Groundwater

**Figure 4:**
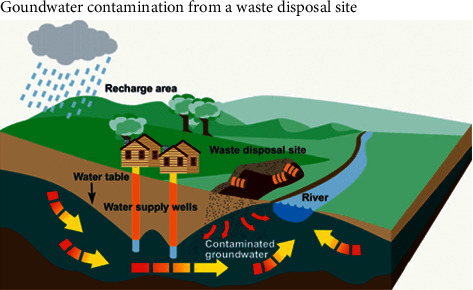
Potential contaminant sources from a waste disposal site (source: Walsh [18], National Academy of Sciences, Washington DC).

Leachate is one of the three most common landfill problems, aside from toxins and greenhouse gases [8]. Leachate production and poor management techniques associated with uncontrolled landfills (especially open dump landfills) pose a significant threat to groundwater. Leachate is the contaminated liquid that drains from the waste material. Leachate is generated when rainwater filters through the waste, and the liquid is leached or drawn out. Chemicals and other constituents from the waste are potent in the leachate [8, 17]. The contaminated liquid collects at the bottom of the landfill where it is typically withdrawn through a collection system. Leachate needs to be properly managed because it can percolate into and contaminate groundwater. One of the major targets of a landfill is to avoid any hydraulic or water-related connection between the waste and the surrounding environment because the primary environmental problem arising from landfills is groundwater contamination [17]. Several hazardous waste materials are deposited into landfills that can decompose, and if not properly managed (open dumping system), will end up in the groundwater (Figures 1 and 4). The toxic products in the landfills can range from industrial solvents to household cleaners. In a study conducted by the USGS on contaminants of emerging concerns (CECs) in landfill leachate, scientists used three analytical methods to determine CEC concentrations: a liquid chromatography-tandem mass spectrometry, a gas chromatography, and a GC/MS method. They found that “final leachate samples contained 101 of the 190 chemicals analyzed for the study, with chemicals present in every final leachate sample collected at levels ranging from as low as 2 nanograms per liter (ng/L) to as high as 17,200,000 ng/L” (2015, p.11). Quality-control samples were collected and analyzed to evaluate bias, accuracy, and precision of CEC concentrations in leachate samples (2015, p.4). Lastly, a one-sided Wilcoxon rank sum test was used to test for any significant differences in the distribution of CEC concentrations between each sample group. The USGS scientists provided a list of all the chemicals found in the leachate [19] (Table 2).

**Figure 5:**
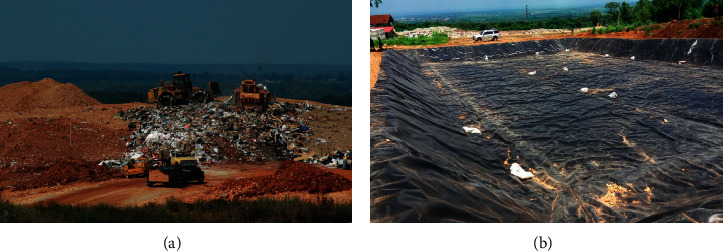
Sites undergoing rehabilitation for municipal solid waste landfill. (a) Reprinted from City of Springfield, https://www.springfieldmo.gov/2331/Solid-Waste-Management-Recycling-and-sanitary-landfill. (b) Reprinted from FABRIMETIRCS PHIL, INC., https://fabphils.com/proj006-bataan-sanitary-landfill-phase-2.

**Figure 6:**
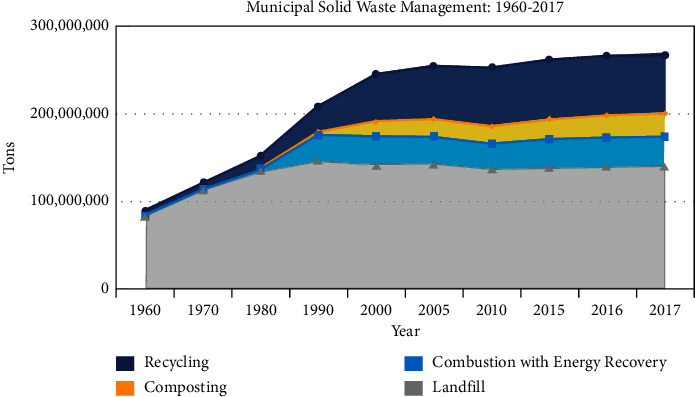
Composition of the US municipal solid waste management between 1960 and 2017 [9].

**Figure 7:**
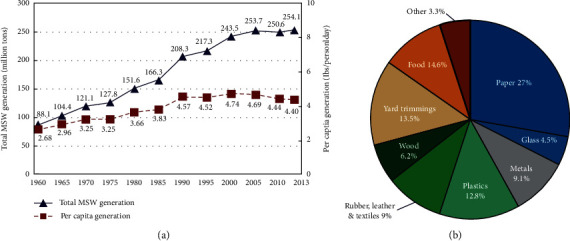
1960–2013 MSW generation (reprinted from EPA Archive, https://archive.epa.gov/epawaste/nonhaz/municipal/web/html/).

Besides the chemicals from household and industrial products, there are also electronic wastes found in landfills that contain lead and mercury. A large percentage of these landfill toxins need to be properly managed in engineered landfills to avoid infiltration of these contaminants into the freshwater housed in underground aquifers. Eventually, the toxins may end up in domestic water and sometimes in the foods that we consume. The contamination can also harm animal and plant life [20]. Research conducted by the United States Environmental Protection Agency [4] reveals that 82% of landfills have leaks that require rehabilitation or remediation for a sustainable system (Figure 5). In a National Geographic article titled *Human Footprint*, Kulpinski [20] states that an “increase in the risk of severe health and environmental implications has been reported in individuals living next to landfill areas in numerous studies.” This calls for comprehensive EIAs to classify the risk index (RI) factor associated with most landfills. There are many public health concerns for people living close to uncontrolled landfills, a large proportion of those health concerns being associated with groundwater contamination from leachate [21]. Capping, the term used to describe covering a landfill, is one of the practices used by companies to prevent further toxic spills and leachate infiltration. While caps are not designed to remove or reduce contaminants, they do isolate and prevent the further spread of those contaminants. This is not an ideal method to eliminate or reduce contamination, but it is an effective short-term solution for containing those contaminants from polluting our lakes, streams, or groundwater [22].

The proper containment and storage of leachate is important, but eventually, the implementation of leachate treatment will be necessary for a long-term solution since the infrastructure holding the leachate can only withstand so long. There are various treatment options available to treat leachate (i.e., biological treatment via biofilters to remove nitrogen and other compounds and physical-chemical processes via oxidation, flocculation, adsorption, etc.); however, it is debatable how cost-effective those treatments are [8]. More research will need to be conducted to determine the most viable and cost-effective method for leachate treatment.

#### 3.1.2. Landfill Gas Generation and Air Pollution

Another important detrimental effect of uncontrolled landfills on the environment is the generation of LFG. The primary LFG emissions are methane and carbon dioxide. However, gases such as hydrogen sulfide and mercury vapor can be emitted at low concentrations, while a mixture of volatile organic compounds (VOCs) comprises approximately 0.5% of gases emitted [4, 23]. The extraction of LFGs is crucial because the gases are an explosive hazard. Furthermore, exposure to these gases can pose a threat to the surrounding population [4].

Companies can extract these gases since landfills are prime candidates for gas recovery and allows companies to execute gas-to-energy projects. This means that the gas generated by the landfill can be taken, converted, and then utilized to generate electricity in the form of heat or steam [21]. For landfill sites that do not have gas-to-energy projects, there are gas flare stations that are used to burn off the flammable gas that is released by pressure relief valves [24]. Although there are other toxic gases emitted from landfills, methane and carbon dioxide are the primary emissions, with methane being the most environmentally damaging [25]. Methane gas is naturally produced during the process of organic matter decay (food, wood, textiles, garden waste, etc.) from bioreactors. The United States Environmental Protection Agency [4] reported that the methane expelled during the decomposition of organic matter, if unmanaged within the landfill, has the potential of trapping solar radiation 20 times more effectively than carbon dioxide. The outcome from the release of these gases from the landfill is increased global temperatures. Aside from methane gas, other household and agricultural chemicals like bleach and ammonia can generate toxic gases that can greatly impact the air quality within the landfill vicinity [26]. Dust, particulate matter, and other nonchemical contaminants can also be expelled into the atmosphere, contributing to poor air quality. As stated above, methane is flammable and LFGs combined with a large amount of landfill waste can easily lead to a fire outbreak if not properly contained. Once fires are ignited, it can be challenging to extinguish them, resulting in further air pollution and destruction of neighboring habitats [27]. Combustion of the landfill worsens the situation, as the burning of the chemicals adds more chemical load into the area. The Environmental Defense Fund [6] states that methane is “84 times more potent than carbon dioxide in the short term.” LFG needs to be constantly monitored and extracted, as the production of toxic gases and odors can significantly impact air quality (United States Environmental Protection Agency (USEPA) [28]).

As part of their Landfill Methane Outreach Program, the USEPA [28] stated that “instead of escaping into the air, LFG can be captured, converted, and used as a renewable energy resource.” By converting LFG, there is a reduction of odor and lower emissions of other hazards associated with LFG. This utilization prevents methane from migrating into the atmosphere, where it could contribute to local smog and global climate change. Besides a reduction in environmental pollution, using LFG as an energy source can also produce jobs and increase revenue [28]. The agency stated that generating power from the emitted methane is a clean, positive action taken by landfills and is a renewable solution (USEPA, 2018) [28]. Chen and Greene [29] of the Natural Resources Defense Council compiled a table of LFG Energy-Use projects (Table 3). Noticeably, LFG has many energy applications.

### 3.2. Human Health Implications

There have been multiple studies that show that residents living in the surrounding communities to uncontrolled landfills have negative health effects, some of which are related to groundwater contamination and air pollution. In a review article by Vrijheid [30] titled *Health Effects of Residence Near Hazardous Waste Landfill Sites*, multiple study sites situated near landfills that may or may not have already experienced environmental issues were observed. The findings were based on frequent concerns from the public of exposure to toxins (whether in the air or water) at a specific site and the health outcomes reported within the surrounding populations. Landfill sites may be a source of airborne chemical contamination through the migration of gases, particles, and chemicals that adhere to dust [30]. This article also states that “other possible routes of exposure include direct contact through the contamination of soil, ground, and surface water or inhalation via pollution of indoor air in the case of evaporation of volatile organic compounds into basements of nearby houses” [30]. If the water used by residents becomes heavily contaminated, other water uses such as bathing may also lead to exposure to evaporated VOCs released from waste [31]. Areas surrounding uncontrolled landfills have increased incidences of health issues such as respiratory issues, skin irritations, gastrointestinal problems, fatigue, headaches, and psychological disorders [30]. Studies on landfill influence on public health are typically only conducted after officials have been notified about an odor being released from a landfill site or after residents have already begun to experience adverse health effects. These are self-reported cases, which show increased effects in populations exposed to waste sites compared with unexposed populations [32]. The review references a study done in Woburn, Massachusetts, where toxic chemicals from an uncontrolled waste disposal site were detected in municipal drinking water wells [33]. Residents of Woburn reported a cluster of leukemia cases in children. These reports were confirmed through hospital and pathology records, and a first study confirmed that this number was significantly higher than expected based on national rates [33]. The parents of the leukemia patients were later interviewed by selecting parents of two exact age and sex-matched well controls—one who lived close to the patient and the other who lived in the distal half of the city. Most of the East Woburn residents reported poor water quality, noting its bad odor, taste, color, and corrosiveness. Findings in these studies are consistent with conclusions made above: poor landfill management leads to air pollution and groundwater contamination, harming both the environment and people.

According to Rushton [21], “reproductive effects associated with landfill sites have been extensively researched and include low birth weight (less than 2500 g), fetal and infant mortality, spontaneous abortion, and the occurrence of birth defects” (p.188). Furthermore, Rushton [21] and Griffith et al. [34] stated that through the use of National Priorities Listing (NPL) of hazardous waste sites developed by the US Environmental Protection Agency, there was an increased frequency of cancers in counties containing hazardous wastes that are not properly stored and disposed of in engineered landfills: particularly implicated are incidences related to gastrointestinal, esophageal, stomach, colon, and rectal cancers. On the other hand, Budnick et al. [35] studied the superfund site in Clinton County, Pennsylvania, and had found that the site was contaminated with the carcinogens *β*-naphthylamine, benzidine, and benzene. According to their study, they found an increased number of bladder cancer deaths among white males in Clinton County and a substantial increase in the number of other cancer deaths in the general population of Clinton and three surrounding counties during the 1970s [35]. However, no specific birth defects were significantly associated with the superfund site in this study.

## 4. Innovative Methods Utilized at Landfills

### 4.1. Reducing Landfill Waste

Though a majority of waste that ends up in landfills could potentially be minimized by implementing practices such as reducing, reusing, and recycling or completely getting rid of the plastic industry, it is just not feasible. Geyer et al. [36] wrote, “as of 2015, approximately 6300 Mt of plastic waste had been generated, around 9% of which had been recycled, 12% was incinerated, and 79% was accumulated in landfills or the natural environment. If current production and waste management trends continue, roughly 12,000 Mt of plastic waste will be in landfills or the natural environment by 2050”. However, there are natural methods of waste disposal that can be utilized upon further investigation. One method is the usage of *Galleria mellonella*, commonly known as wax moth caterpillars. Polyethylene (PE) is a form of plastic that cannot biodegrade, as it is held together by C-C bonds [37]. In a study conducted by Bombelli et al. [37], the researchers placed a PE film next to the caterpillars and found destruction of the plastic within 40 minutes. The researchers reported that after placing 100 caterpillars in contact with a plastic bag for 12 hours, there was a 92 mg reduction in the mass of the bag [37]. To account for the possibility that mechanical action was solely responsible for the PE breakdown, worm homogenates were smeared on and left in contact with the PE films. Gravimetric analysis of the treated samples confirmed significant mass loss of 13% PE over the span of 14 hours compared with the untreated samples [37]. The *G. mellonella* caterpillars achieved this by breaking down the chemical bonds that the PE consists of according to FTIR analysis. However, further research is necessary to determine whether the hydrocarbon-digesting activity of *G. mellonella* is derived from the organism or from enzymatic activities of its intestinal flora [37, 38]. Hopefully, further research and understanding of the *G. mellonella* caterpillars will one day be implemented on a larger scale to combat landfill waste.

### 4.2. Reducing Gas Emissions

Other approaches considered more sustainable with regard to airspace, processes, control, and product utilization with minimal negative effects on the environment and human health include integrating engineered landfills with anaerobic bioreactors or aerobic biocells. These systems will help reduce methane gas emissions by capturing, purifying, and redirecting the gas to be used toward energy projects.

As mentioned in an earlier section, methane is one of the primary greenhouse gases emitted from landfills. A journalist at the Technical University of Denmark, Jensen [39], writes that compared with carbon dioxide, methane is 25 times more detrimental. Thus, it is important to mitigate its effects. A common method utilized to reduce the release of methane is using soil to cover up landfill sites. However, not all gas is contained with this method [39]. In a pilot study conducted by Denmark researchers Schuetz et al. [40], a new biocover technology was utilized to achieve just this. This biocover technology functions by “sealing the surfaces of old landfills to prevent methane from penetrating them. Instead—by means of a gas drainage system—the gas is distributed through so-called ‘biowindows,' which are most comparable to a compost bed. Here, the natural microorganisms of the compost transform methane into CO_2_ [39].”After implementing this technology at a Danish landfill site, they found that they were able to reduce methane production from 10 kg/hr to just 1 kg/hr [39]. This technology has proven effective in managing one greenhouse gas and has the potential to be utilized worldwide, greatly reducing methane emissions at landfill sites.

## 5. Discussion

Determining and assessing the risks posed by landfills is essential to relieving their direct effects on the environment. It is important to stress the issue of proper landfill management as a means of decreasing leachate and LFG contamination. Although landfills are environmentally straining, some actions can be taken to alleviate negative impacts. When a landfill reaches the end of its lifespan and can no longer collect any more waste, the waste management company will cap it [4]. Once closed, the landfill goes into the postclosure process where the company is responsible for managing that landfill for at least the next 30 years. Management is necessary as leachate and gas do not stop being produced. Once a landfill has been shut down, that land does not have many alternative uses [4]. However, companies are beginning to develop solar farms on top of landfills, which helps generate revenue. Furthermore, the multiple tax incentives provide more reason for companies to engage in the renewable energy industry.

Another important step that can be taken is to enforce federal regulation of landfills, which would ensure that the construction of landfills is well engineered and properly managed. Enforcement can significantly lessen the impacts a landfill has on the quality of soil, air, and water [4]. Landfills that are well designed and properly operated ensure compliance with environmental preservation requirements and ultimately ensure that the environment is contaminant-free [28]. Proper construction and maintenance also ensures that landfills are not located in environmentally sensitive areas and are incorporated with on-site environmental monitoring systems that track signs of gas release and groundwater contamination [28]. Landfill management will need to focus on designing and operating sustainable landfills. Similarly, communities also need to be a part of this sustainable future by focusing on decreasing their waste generation and thereby effectively limiting the negative effects of landfills [9].

## 6. Conclusion

According to the United States Environmental Protection Agency [4], the following steps can be taken to reduce our waste production and protect the environment: (1) respect the planet and all of its living and nonliving components, (2) rethink our consumption needs and avoid spending money on unnecessary things, (3) reduce wastage and waste accumulation, and (4) reuse and recycle products when possible. According to Annenberg Foundation [41], we can eventually reuse or recycle more than 70 percent of landfilled wastes, as the majority comprises valuable materials such as glass, metal, and paper. By reusing and recycling those materials, the demand for original natural sources of these materials can be reduced, and this can potentially eliminate severe environmental, economic, and public health issues. All in all, with our increasing population, waste generation will also increase, so it is crucial to recognize and mitigate the issues of leachate production and LFG generation to protect the environment and human health. Waste management companies need to enforce strict landfill regulations and people need to take it upon themselves to reduce their waste generation, which will reduce both the toxicity and volume of waste that ends up in landfills. However, in the time being, further studies need to be conducted to achieve greater insight into *G. mellonella* caterpillars and biocover technology, as both methods have proven effective and could be crucial in reducing the detrimental effects of landfills and landfill waste worldwide.

## Figures and Tables

**Table 1:** Some specific criteria for rapid risk assessment of landfills [16].

SI. no.	Attribute (site-specific criteria)	Attribute weightage	Sensitivity index			
			0.0–0.25	0.25–0.5	0.5–0.75	0.75–1.0
						
1.	Distance from nearest water supply source (m)	69	>5000	2500–5000	1000–2500	<1000
2.	Depth of filling of waste (m)	64	<3	3–10	10–20	>20
3.	Area of dumpsite (ha)	61	<5	5–10	10–20	>20
4.	Groundwater depth (m)	54	>20	10–20	3–10	<3
5.	Permeability of soil (1 × 10^−6^ cm/s)	54	<0.1	1–0.1	1–10	>10
6.	Groundwater quality	50	Not a concern	Potable	Potable if no alternative	Nonpotable
7.	Distance to critical habitats such as wetlands and reserved forest (km)	46	>25	10–25	5–10	<5
8.	Distance to the nearest airport (km)	46	>20	10–20	5–10	<5
9.	Distance from surface water body (m)	41	>8000	1500–8000	500–1500	<500
10.	Type of underlying soil (% clay)	41	>50	30–50	15–30	0–15

**Table 2:** List of 101 chemicals found in leachate samples from 22 landfills in the US.

Chemical^a^	CASRN^b^	RL range (ng/L)	Frequency (T)	Maximum (ng/L)	Detection median^c^ (ng/L)	Primary chemical use
Diazepam (1)	439-14-5	2-44	5	E 42. 1	E 42. 1	Antianxiety, sleep aid, anticonvulsant
Diltiazem (1)	42399-41-7	10-204	5	12.0	12.0	Calcium channel blocker
Erythromycin (1)	114-07-8	53-1060	5	204	204	Antibiotic
Fluconazole (1)	86386-73-4	71-1420	50	1520	180	Triazole antifungal
Glipizide (1)	29094-61-9	35-692	5	155	155	Antidiabetic
Glyburide (1)	10238-21-8	4-79	9	25.8	24.4	Antidiabetic
Loperamide (1)	53179-11-6	11-230	5	47.4	47.4	Antidiarrheal
Lorzepam (1)	846-49-1	116-1160	5	E 4820	E 4820	Antianxiety
Meprobamate (1)	57-53-4	86-1720	36	E 1530	467	Carbamate derivative, anxiolytic
Metaxalone (1)	1665-48-1	15-312	41	1710	303	Muscle relaxant
Metformin (1)	657-24-9	13-262	41	838	395	Antidiabetic
Methadone (1)	76-99-3	7-152	9	1932	981	Synthetic opioid, analgesic
Methocarbamol (1)	532-03-6	9-174	36	1210	144	Muscle relaxant
Methotrexate (1)	59-05-2	52-1050	9	315	254	Antifolate
Metoprolol (1)	51384-51-1	28-550	14	E 461	E 423	Antihypertensive
Nadolol (1)	42200-33-9	81-1620	9	E 319	238	Beta blocker
Nizatidine (1)	76963-41-2	19-380	5	25.3	25.3	Acid inhibitor
Oseltamivir (1)	196618-13-0	15-292	9	E 147	E 83.3	Antiviral
Paroxetine (1)	61869-08-7	21-412	5	E 73.3	E 73.3	Antidepressant
Penciclovir (1)	39809-25-1	40-400	5	E 2140	E 2140	Antiviral
Pentoxifylline (1)	6493-05-6	9-187	23	2841	856	Circulation enhancer (peripheral blood flow)
Phendimetrazine (1)	634-03-7	31-622	5	E 1110	E1110	Appetite suppressant
Phenytoin (1)	57-41-0	188-3760	32	2410	274	Antiepileptic
Quinine (1)	130-95-0	79-1600	5	E 284	E 284	Antimalarial, flavorant, mild antipyretic and analgesic
Sulfadimethoxine (1)	122-11-2	65-1310	18	E 401	183	Antibiotic
Sulfamethizole (1)	144-82-1	104-2080	5	861	861	Antibiotic
Thiabendazole (1)	148-79-8	4-82	55	1770	211	Parasiticide, fungicide
Tramadol (1)	27203-92-5	15-302	55	1490	279	Opiate
Triamterene (1)	396-01-0	5-105	18	14.9	12.7	Diuretic
Valacyclovir (1)	124832-26-4	163-3260	5	E 765	E 765	Antiviral
Venlafaxine (1)	93413-69-5	5-90	5	168	168	Antidepressant
Warfarin (1)	81-81-2	6-121	36	E 70	23.0	Anticoagulant, rodenticide
*Steroid hormones*						
cis-Androsterone (2)	53-41-8	0.8	23	125	72.3	Natural androgen
Equilenin (2)	517-09-0	1	5	18	18	Natural equine estrogen, hormone replacement therapy
Estriol (2)	50-27-1	2	9	6.50	5.01	Natural estrogen
Estrone (2)	53-16-7	0.8	23	145	18.1	Estradiol degradate
Norethindrone (2)	68-22-4	0.8	5	30. 1	30.1	Synthetic progestin
*Household chemicals*						
Acetophenone (3)	98-86-2	4000	23	E 63800	15800	Fragrance and/or flavorant
Benzophenone (3)	119-61-9	400-1600	32	E 7310	2690	Fixative for perfumes and soaps
Bisphenol A (BPA) (2)	80-05-7	100	77	E 17200000	E 45400	Component of plastics and thermal paper
Camphor (3)	72-22-2	400	55	E 342000	62400	Fragrance and/or flavorant
d-Limonene (3)	5989-27-5	1600	5	E 3400	E 3400	Pesticide, fragrance in aerosols
Galaxolide (3)	1222-05-5	200	14	E 928	302	Polycyclic musk fragrance
Isoquinoline (3)	119-65-3	400	5	801	801	Fragrance and/or flavorant
Menthol (3)	1490-04-6	3200	18	82900	27800	Flavorant
N, N-diethyltoluamide (DEET) (3)	134-62-3	400	68	E 431000	45500	Insect repellent
Skatol (3)	83-34-1	400	23	31900	8200	Fragrance
Tri(2-chloroethyl)phosphate (3)	115-96-8	6400	27	9100	8100	Plasticizer, flame retardant
Tri(dichlorisopropyl)phosphate (3)	13674-87-8	1600	9	E 2390	E 2070	Flame retardant
Tributylphosphate (3)	126-73-8	640	45	7770	2000	Antifoaming agent, flame retardant
*Industrial chemicals*						
1,4-Dichlorobenzene (3)	106-46-7	400	32	2830	E 797	Moth repellent, fumigant, deodorant
1-Methylnaphthalene (3)	90-12-0	400	18	2260	983	Component of petroleum
2,6-Dimethylnaphthalene (3)	581-42-0	400	5	421	421	Component of diesel/kerosene
2-Methylnaphthalene (3)	91-57-6	400	9	2840	1900	Component of petroleum
3,4,Dichlorophenyl isocyanate (3)	102-36-3	200	5	E 1010	E 1010	Industrial chemical intermediate
4-Cumylphenol (3)	599-64-4	400	18	E 12800	E 10000	Plasticizer, flame retardant
4-Nonylphenol (3)	84852-15-3	200	32	E 83200	E 18500	Nonionic detergent degradate
4-Nonylphenol diethoxylate (3)	26027-38-2	2000	18	E 146000	24500	Nonionic detergent degradate
4-Tert-octylphenol (3)	140-66-9	400	55	E 6870	E 1860	Nonionic detergent degradate
4-Tert-octylphenol diethoxylate (3)	2315-61-9	2000	5	47000	47000	Nonionic detergent degradate
4-Tert-octylphenol monoethoxylate (3)	2315-67-5	2000	5	15300	15300	Nonionic detergent degradate
5-Methyl-1H-benzotriazole (3)	136-85-6	3200	18	E 6480	E 5820	Antioxidant in antifreeze and deicers
Anthracene (3)	120-12-7	200	27	1570	631	Component of tar, diesel, or crude oil
Anthraquinone (3)	84-65-1	400	14	E 691	E 532	Dye/textiles, seed treatment, bird repellent
Diethyl phthalate (3)	84-66-2	2000	18	E 14100	6500	Plasticizer for polymers and resins
Fluoranthene (3)	206-44-0	200	5	E 430	E 430	Component of coal tar and asphalt
Isopropylbenzene (3)	98-82-8	400	18	1110	964	Fuels and paint thinner
Methyl-1H-benzotriazole (1)	29385-43-1	141-2820	59	E 9660	1310	Corrosion inhibitor
Naphthalene (3)	91-20-3	200	55	17300	598	Fumigant, component of gasoline
Para-cresol (3)	106-44-5	800	32	1580000	117000	Wood preservative
Phenanthrene (3)	85-01-8	200	23	3600	358	Explosives, component of tar and diesel fuel
Phenol (3)	108-95-2	1600	27	E 1190000	E 98500	Disinfectant
*Nonprescription pharmaceuticals and degradates*						
Acetaminophen (1)	103-90-2	7-143	41	42600	5300	Analgesic, antipyretic
Caffeine (1)	58-08-2	900-1810	32	3360	1340	Stimulant
Cimetidine (1)	51481-61-9	27-556	18	1085	211	Histamine H2-receptor antagonist
Cotinine (1)	486-56-6	18-127	86	E 30400	E 597	Nicotine degradate
Dextromethorphan (1)	125-71-3	8-64	18	204	70.3	Cough suppressant
Diphenhydramine (1)	147-24-0	6-116	9	24	15.7	Antihistamine
Fexofenadine (1)	83799-24-0	20-398	14	E 252	E 237	Antihistamine, terfenadine degradate
Lidocaine (1)	137-58-6	15-304	91	E 47900	5380	Local anesthetic
Loratadine (1)	79794-75-5	7-139	5	E 202	E 202	Antihistamine
Nicotine (1)	54-11-5	1160	23	E 43800	E 6080	Alkaloid stimulant
Piperonyl butoxide (1)	51-03-6	3-161	23	E 238	35.7	Pesticide synergist
Pseudoephedrine (1)	90-82-4	11-222	45	E 6200	2150	Appetite suppressant, decongestant, stimulant
*Pesticides and degradates*						
Atrazine (1)	1912-24-9	19-388	9	507	466	Herbicide
Carbaryl (3)	63-25-2	600	5	E 2530	E 2530	Insecticide
*Plant and animal sterols*						
3-Beta-coprostanol (3)	360-68-9	200	59	176000	7980	Fecal indicator
Beta-sitosterol (3)	83-46-5	24000	5	190000	190000	Phytoestrogen
Cholesterol (3)	57-88-5	200	73	32300	7300	Plant and animal sterol
Stigmastanol (3)	19466-47-8	17000	9	164000	143000	Phytosterol
*Prescription pharmaceuticals and degradates*						
1-Hydroxy-amitriptyline (1)	64520-05-4	8-166	5	415	415	Amitriptyline degradate
Abacavir (1)	136470-78-5	22-444	5	38.1	38.1	Antiviral, reverse transcriptase inhibitor
Acyclovir (1)	59277-8403	22-444	27	2720	582	Antiviral, reverse transcriptase inhibitor
Albuterol (1)	18559-94-9	6-121	18	377	268	Bronchodilator
Amphetamine (1)	300-62-9	8-163	45	11900	614	Psychostimulant
Antipyrine (1)	60-80-0	116-2320	23	E 1060	189	Analgesic, antipyretic
Atenolol (1)	29122-68-7	13-266	32	1042	E 178	Beta blocker
Bupropion (1)	34841-39-9	17-356	5	38.8	38.8	Antidepressant
Carbamazepine (1)	298-46-4	4-83	77	E 810	165	Anticonvulsant and mood stabilizer
Carisoprodol (1)	78-44-4	13-250	82	E 3060	322	Muscle relaxant
Desvenlafaxine (1)	93413-62-8	7-150	7	E 656	225	Venlafaxine degradant

^a^Value in parentheses indicates method: (1) = liquid chromatography-tandem mass spectrometry (LC-MS/MS) pharmaceuticals; (2) = gas chromatography-tandem mass spectrometry (GC-MS/MS) steroid hormones; (3) = gas chromatography/mass spectrometry (GC/MS) household/industrial chemicals. ^b^Chemical abstracting service report number. ^c^Median of detected concentrations. CEC = contaminant of emerging concern; E = flagged due to concentration being less than the RL or greater than the highest point on calibration curve; RL = reporting limit; Maximum = maximum concentration. *Note.* Reprinted from Landfill Leachate as a Mirror of Today's Disposable Society: Pharmaceuticals and Other Contaminants of Emerging Concern in Final Leachate from Landfills in the Conterminous United States, by Masoner, J.R., Koplin, D.W., Furlong, E.T., Cozzarelli, I.M., and Gray, J.L. (2015b). Retrieved from https://setac.onlinelibrary.wiley.com/doi/abs/10.1002/etc.3219 copyright 2015 by *Environmental Toxicology and Chemistry*.

**Table 3:** Different uses for LFG energy.

Landfill-gas energy use		Number of projects
Electric		
	Reciprocating engines	187
	Gas turbines	31
	Other	25
	All electric	243
Direct		
	Boilers	29
	Direct thermal and leachate evaporation	47
	Other	31
	All direct	107
Total		350
All landfills		2,239

*Note.* Reprinted from Is Landfill Gas Green Energy?, by Chen and Greene [29]. Retrieved from https://www.nrdc.org/sites/default/files/lfg.pdf copyright 2003 by the Natural Resources Defense Council.

## Data Availability

No data set was generated in this review article. References are provided for the data used in this review.
